# Heroin Overdose-Related Child and Adolescent Hospitalizations: Insight on Comorbid Psychiatric and Substance Use Disorders

**DOI:** 10.3390/bs9070077

**Published:** 2019-07-13

**Authors:** Uwandu Queeneth, Narmada N. Bhimanadham, Pranita Mainali, Henry K. Onyeaka, Amaya Pankaj, Rikinkumar S. Patel

**Affiliations:** 1 Department of Psychiatry, Maastricht University, 4–6, 6211 LK Maastricht, The Netherlands; 2 Department of Public Administration, Drake University, Des Moines, IA 50311, USA; 3 Department of Psychiatry, Washington DC VA Medical Center, Washington, DC 20422, USA; 4 Harvard School of Public Health, Boston, MA 02115, USA; 5 Jawaharlal Institute of Postgraduate Medical Education and Research (JIPMER), Puducherry 605006, India; 6 Department of Psychiatry, Griffin Memorial Hospital, Norman, OK 73071, USA

**Keywords:** heroin overdose, opioid abuse, adolescents, emergency visits, hospitalization, substance use, epidemiology

## Abstract

Objective: To evaluate the association between psychiatric comorbidities, substance use disorders and heroin overdose-related hospitalizations (HOD). Next, to understand the demographic trend of HOD hospitalizations and comorbidities. Methods: Using the Nationwide Inpatient Sample (NIS), we included 27,442,808 child and adolescent hospitalizations, and 1432 inpatients (0.005%) were managed primarily for HOD. The odds ratio (OR) of the association of variables in HOD inpatients were measured using a logistic regression model. Results: Adolescents had 56 times higher odds (95% CI 43.36–73.30) for HOD-related hospitalizations compared to 4.6% children under 11 years. About three-fifth of the HOD inpatients were male, and they had 1.5-fold higher odds (95% CI 1.30–1.64) compared to 43% females in the study population. Whites were considerably higher in proportion (81%) than other race/ethnicities. A greater portion of HOD inpatients (40%) were from high-income families. Most common comorbid psychiatric disorders were mood (43.8%) and anxiety (20.4%). The prevalent comorbid substance use disorders were opioid (62.4%), tobacco (36.8%) and cannabis (28.5%) use disorders. Conclusion: HOD-related hospitalizations were predominant in males, White and older adolescents (12–18 years). Prescription opioids are the bridge to heroin abuse, thereby increasing the vulnerability to other substance abuse. This requires more surveillance and should be explored to help reduce the heroin epidemic in children.

## 1. Introduction

Opioids overdose continues to be a public health emergency globally. In the past 18 years, nearly 8986 children and adolescents have died from illicit opioid use, particularly heroin, in the US [1]. Heroin is an extremely addictive opioid and has no acceptable medical indications; however, an estimated number of 828,000 people over 12 years of age used heroin in the US within the past year, and the annual rates were highest in adolescents aged 15–19 [2]. Approximately 20% of emergency department visits attributed to illegal substance abuse were due to heroin, and each year these numbers are rising [2]. Studies reveal that many heroin abusers switched from prescription opioids to heroin after becoming addicted due to the fact that heroin has become more accessible and less expensive than prescription opioids [3]. This raising accessibility may have caused an increase in overdoses amongst this population [3].

Adolescents at an increased risk of heroin abuse include but are not limited to those with a history of mental illness, other drug abuse such as prescription opioids, and family members who abuse the same drugs [4]. Lower-risk adolescents include those with higher academic performances and those whose guardians strongly discourage substance abuse [4].

There are overlapping factors associated with substance abuse and mental illnesses. Heroin addiction co-occurs with some other psychiatric illnesses, including depression, anxiety disorder and bipolar disorder [5]. Furthermore, amongst heroin abusers, there is a trend of concurrent addiction and abuse of multiple substances, especially cannabis, cocaine, benzodiazepines and alcohol [5]. These factors have significantly worsened their mental health and increased the number of emergency visits and drug overdoses amongst this population [6].

There is a notable variation in patterns in heroin overdose based on race, gender, age, and socioeconomic status, for instance, non-Hispanic Whites are mostly impacted, compared to other races [7]. Additionally, the overdose rates in males aged 15–19 are reported to be constantly high as compared to their female counterparts [8]. Furthermore, people living in large metropolitan cities had higher rates, as did those from a lower socioeconomic status [1].

We used the inpatient data from US hospitals to evaluate the prevalence of hospital admissions for heroin opioid overdose (HOD) in child and adolescent inpatients, and to measure the association between psychiatric comorbidities, substance use disorders and HOD-related hospitalization. Next, we analyzed the trend of emergency admissions and HOD-related hospitalization by demographic and comorbidities from 2010 to 2014 in US hospitals.

## 2. Methods

### 2.1. Data Source

We used the Nationwide Inpatient Sample (NIS) from the Healthcare Cost and Utilization Project (HCUP), which is the largest hospital-based dataset in the United States. The NIS is comprised of patient discharge diagnoses information (clinical and non-clinical) from 4400 hospitals across 45 states in the US. We applied the discharge weights (DISCWT) present in the NIS to the unweighted data to get national representative estimates [9]. Diagnostic information in the NIS is identified using the International Classification of Diseases, Ninth Edition, Clinical Modification (ICD-9-CM) codes [10].

### 2.2. Inclusion Criteria and Variables

We conducted a retrospective analysis of the NIS (2010 to 2014) to identify non-elective hospital admissions for the child and adolescent patients (age ≤ 18) with a primary ICD-9-CM discharge diagnosis for HOD (965.02). Adult patients (>18 years) and elective admissions were excluded from our study data.

Demographic variables, including age, sex, race and median household income, were evaluated in this study [9]. Comorbid psychiatric disorders (anxiety disorder, mood disorders, psychotic disorders, and suicidality) and substance use disorders (tobacco, cannabis, opioid, amphetamine, and cocaine) were identified using the ICD-9-CM diagnosis codes and HCUP Clinical Classification Software (CCS) codes [9,11]. Suicidality was identified by exploring the patient records for co-diagnoses of suicidal and intentional self-inflicted injury (CCS code 662).

### 2.3. Statistical Analysis

We utilized a bivariate descriptive analysis to compare demographics and comorbid psychiatric and substance use disorders in patients with HOD and without HOD (non-HOD), followed by binomial logistic regression analyses to evaluate the demographic predictors and associated comorbidities for HOD-related hospitalization. Next, we studied the trends for HOD-related hospitalization from 2010 to 2014. We used a linear-by-linear association and descriptive statistics for the categorical data. The analyses were conducted using Statistical Package for the Social Sciences (SPSS) version 25 (IBM Corp., Armonk, NY, USA) with the statistical significance set at <0.05.

### 2.4. Ethical Approval

Unique subject identifiers (KEY_ID) were used to secure the patient information [9]. The use of the publicly available de-identified NIS data of the HCUP [10], according to the Department of Health and Human Services, does not require approval from an Institutional Review Board.

## 3. Results

We analyzed 27,442,808 child and adolescent hospitalizations, and 1432 inpatients (0.005%) were managed primarily for HOD. Adolescents had 56 times higher odds (95% CI 43.36–73.30) for HOD hospitalization, compared to 4.6% children under 11 years. About three-fifth of the HOD inpatients were male, and they had 1.5-fold higher odds (95% CI 1.30–1.64), compared to 43% females in the study population. With regards to race, Whites were considerably higher in proportion (81%) than other race/ethnicities, as shown in Table 1. A greater portion of HOD inpatients (40%) were from high-income families with a median household income above the 76th percentile, and the likelihood for HOD hospitalization increased with a rising income.

The most prevalent comorbid psychiatric disorders were mood (43.8%) and anxiety (20.4%) disorders. In the adjusted regression model, children with anxiety disorders have 1.2-fold higher odds of an association (95% CI 1.05–1.40) with HOD hospitalization. The most prevalent comorbid substance use disorders were opioid (62.4%), tobacco (36.8%) and cannabis (28.5%) use disorders. After controlling with demographic confounders and comorbid psychiatric disorders, the higher odds of association with HOD hospitalization were seen with children with comorbid opioid (OR 195.7, 95% CI 167.71–228.32), tobacco (OR 1.7, 95% CI 1.51–1.98) and cocaine (OR 1.5, 95% CI 1.12–1.76) use disorders, as shown in Table 1.

A trend study was conducted to see the differences in HOD child and adolescent inpatients from 2010 to 2014, as shown in Table 2. There was a notable decreasing trend by 18.7% in the HOD hospitalization from 289 inpatients in 2010 to 235 in 2014. The proportion of HOD hospitalization decreased statistically significantly from 94.5% in 2010 to 88.7%, and it increased by 87.5% in children under 11 years (16 inpatients in 2010 to 30 inpatients in 2014). Over the five-year period, HOD hospitalization was prevalent in males, Whites, and individuals from high income families, and there were no statistically significant changes during the study period by sex, race and median household income. Furthermore, among psychiatric comorbidities, only suicidality significantly increased from 1.7% in 2010 to 5.7% in 2014. Among comorbid substance use disorders, there was a statistically significant rise of opioids, tobacco and cocaine, as shown in Table 2.

## 4. Discussion

Our findings revealed that a higher socioeconomic status (SES), male gender, and White race were strongly associated with HOD, and these findings are consistent with a study using the National Violent Death Reporting System (2016) on opioid overdose [12]. Caucasian HOD inpatients were reportedly (81%) consistent with our findings, and the race difference in HOD has also been reported by several studies [9,13]. Furthermore, adolescents (aged 12–18 years) were more affected compared to the younger age group.

Using the NIS data, we found that HOD inpatients not only abuse heroin but that there is also a comorbid abuse or dependence of other substances like cocaine, tobacco, cannabis, and amphetamine. However, a study by Rodríguez-Llera et al. [14] found cocaine to be most abused substance, whereas in our study tobacco was more abused than cocaine. These findings are consistent with a study demonstrating that the combination of drug types in drug overdose deaths is a common occurrence across the US [15,16]. Psychiatric comorbidities were also observed amongst the HOD inpatient population; they included mood disorders, anxiety disorders, suicide, and psychotic disorders. The Rodriguez et al. [14] study findings were consistent with our data and further revealed that, amongst heroin abusers, women were at a high risk of comorbid mood disturbances when compared to men. Furthermore, a lower percentage of their study suffered major depressive and borderline personality disorders [14].

Furthermore, our study shows that substance abuse is related to the family SES. We found that a good number of HOD inpatients (40%) are from a higher SES. According to a study by Trim et al. [17], adolescents who are more likely to abuse substances are ones who live in an affluent neighborhood. This contributes to a high substance abuse likely because there is less supervision of children in these areas. Additionally, there is more exposure to peers who abuse substances [17]. Affluent children are also at an increased risk for other substances, such as alcohol, during their transition to adulthood [17]. Non-Hispanic males were at a relatively higher risk compared to other races [18,19] for substance use; however, non-Hispanic Black patients account for a greater number of casualties [18].

Our study provides a unique picture of the trends in the US hospitals from 2010 to 2014 of HOD-related hospitalizations in the child and adolescent population. Our findings revealed that comorbid cocaine and tobacco use disorders had significantly increased over the past five years in HOD inpatients. This may be due to an increase in the accessibility to these substances [3]. We found that the suicide rate in HOD inpatients had increased significantly over the past years from 1.7% in 2010 to 5.7% to 2014. This increased rate could be the result of a combination of other illicit drugs, as most heroin addicts may abuse other substances [20]; it is therefore of the utmost importance to develop de-addiction programs to reduce this increasing poly-substance abuse in the HOD child and adolescent population. According to a study by Dragisic et al. [21], intravenous heroin abuse is a significant risk factor for suicidality among addicts. However, as per a study by Gaither et al., the majority of the deaths among drug addicts were unintentional [18], comorbid psychotic disorders declined over the years from 3.5% in 2010 to 0% in 2014, and, conversely, HOD inpatients with comorbid mood disorders increased from 40.5% to 45.3%, though this was not statistically significant.

According to the Centers for Disease Control and Prevention, people addicted to prescription opioids are 40 times more likely to abuse heroin [22]. This can be mitigated by reducing the access of prescription opioids, as most studies revealed that most heroin abusers transitioned from prescribed opioids. Therefore, improving physicians’ prescription practices is a critical way to reduce the heroin addiction problem. Furthermore, pediatric providers should discuss the importance of safe medication storage with guardians, as this is another essential way of reducing this problem [23]. Parents or guardians who are on prescribed opioid should be advised to keep these medications away from their children’s reach [23]. Additionally, another way to reduce the overdose prevalence is by medication-assisted treatment (MAT), i.e., a combined use of methadone, buprenorphine, or naltrexone for heroin addicts [20]. Nevertheless, buprenorphine is associated with misuse [24], leading to cardiovascular and dermatological complications [25,26]. Furthermore, counseling and cognitive behavioral therapies and counseling are also recommended as strategies for harm reduction amongst this population [22].

Younger children are more vulnerable to the accidental ingestion of opioids [27]. Certain strategies should be developed and also implemented to curb heroin overdoses in the pediatric population. Child-serving agencies, i.e., child welfare or child protection services, should provide services and treatments to impacted children and their families, which can reduce long-term catastrophe and prevent fatal outcomes [28]. Children whose guardians or parents abuse heroin, treatment programs such as social rehabilitation, parental coaching and mentoring, and cognitive behavior parental skills should be explored; furthermore, physicians should work closely to oversee parents and their child’s progress [28].

There were several limitations to our study. First, these findings may be underreported, thereby making them unrepresentative of the real burden of HOD across the US due to the nature of the administrative database based on ICD-9 codes that was used in this study. Second, we examined pooled data and year-by-year trend data, though we cannot rule out the possibility that outpatients, and re-hospitalizations across different healthcare settings, may not be represented. We used the NIS, which enabled us to get a national representative sample of HOD inpatients, and the large sample size also increased the power of our study. We compared the trends over five years, which gave us a broader picture and increased the external validity. Furthermore, the findings in this study are useful for understanding the burden of HOD, which can help to adequately and strategically devise specific intervention plans to curb the problem.

## 5. Conclusions

The heroin epidemic is on the rise in the US and has gained much attention, both nationally and internationally. Drug overdose has been noted to be the leading cause of death amongst heroin abusers. Recently, there have been several studies that have been carried out to investigate this problem, compared to decades ago when there was a dearth of information. To reduce the burden of heroin overdose cases in the pediatric population, adequate measures must be taken: both short-term measures and long-term interventions. One approach to reducing the heroin crisis is to improve the access to care for at-risk children of all age groups; additionally, expanding services and the provider’s capacity can be effective ways that would allow affected children and their families to receive the necessary services and support. More inventive options would be needed in the long term, such as decreasing risk factors for multi-drug abuse, and also providing take-home treatment drugs like naloxone to individuals at risk. The implementation of these interventions is demanding but important, and it must be reviewed in addressing the burden of overdose.

## Figures and Tables

**Table 1:** Suspected risk factors for heroin opioid overdose-related hospitalizations.

Variable	Non-HOD (%)	HOD (%)	OR	95% CI	*p* Value
Total inpatients	27,442,808	1432	-	-	-
Age at admission, in years					
<11	88.9	4.6	Reference		
12–18	11.	95.4	56.38	43.36–73.30	<0.001
Sex					
Male	51.0	57.2	1.46	1.30–1.64	<0.001
Female	49.0	42.8	Reference		
Race					
White	51.3	81.0	Reference		
Black	15.8	1.9	0.18	0.12–0.27	<0.001
Hispanic	21.4	10.8	0.80	0.66–0.97	0.023
NA/Asian	11.5	6.3	0.97	0.77–1.23	0.819
Median household income, in percentile					
0–25th	29.0	12.6	Reference		
26th–50th	25.1	20.9	1.41	1.15–1.72	0.001
51st–75th	24.6	26.5	1.77	1.47–2.14	<0.001
76th–100th	21.3	40.0	2.38	1.99–2.84	<0.001
Comorbid psychiatric disorders					
Anxiety disorders	1.4	20.4	1.21	1.05–1.40	0.010
Mood disorders	3.0	43.8	1.06	0.930–1.21	0.386
Suicidality	1.0	3.1	0.19	0.14–0.25	<0.001
Psychotic disorders	0.3	1.0	0.42	0.25–0.70	0.001
Comorbid substance use disorders					
Tobacco	0.6	36.8	1.73	1.51–1.98	<0.001
Cannabis	0.6	28.5	0.83	0.72–0.96	0.011
Opioid	0.1	62.4	195.68	167.71–228.32	<0.001
Amphetamine	0	7.1	1.03	0.81–1.32	0.814
Cocaine	0	9.4	1.45	1.12–1.76	<0.001

The proportion of non-HOD and HOD patients were obtained using cross tabulation and the Pearson Chi-Square (χ^2^) test. Significant *p* values ≤ 0.05 at a 95% Confidence Interval. The odds ratio was generated by a binomial logistic regression model and was adjusted for all variables mentioned. POD: heroin opioid overdose; NA: Native American; OR: odds ratio; CI: confidence interval. HOD: heroin-overdose.

**Table 2:** Trends in child and adolescent inpatients with heroin opioid overdoses.

Variable	2010 (%)	2011 (%)	2012 (%)	2013 (%)	2014 (%)	*p* Value
Total inpatients	289	258	295	305	235	-
Age at admission, in years						
<11	5.5	0	3.3	3.2	11.3	0.001
12–18	94.5	100.0	96.7	96.8	88.7	
Sex						
Male	60.9	61.2	49.2	57.1	58.5	0.336
Female	39.1	38.8	50.8	42.9	41.5	
Race						
White	80.9	79.1	77.2	91.2	75.0	0.726
Black	0	4.0	3.5	1.8	0	
Hispanic	13.0	8.4	12.3	5.3	15.9	
NA/Asian	6.1	8.4	7.0	1.8	9.1	
Median household income, in percentile						
0–25th	12.5	12.3	13.6	17.7	5.8	0.294
26th–50th	21.9	30.4	13.6	16.1	25.0	
51st–75th	24.3	22.5	28.8	25.8	30.8	
76th–100th	41.3	34.8	44.1	40.3	38.5	
Comorbid psychiatric disorders						
Anxiety disorders	20.8	18.2	16.4	25.4	20.8	0.329
Mood disorders	40.5	40.7	49.2	42.9	45.3	0.221
Suicidality	1.7	1.9	4.9	1.6	5.7	0.032
Psychotic disorders	3.5	0	1.6	0	0	<0.001
Comorbid substance use disorders						
Tobacco	28.7	40.3	32.8	44.4	37.7	0.009
Cannabis	28.8	22.9	32.8	30.2	26.4	0.773
Opioid	46.9	55.6	65.6	74.6	67.9	<0.001
Amphetamine	5.2	6.6	6.6	11.1	5.7	0.201
Cocaine	7.3	7.4	9.8	11.1	11.3	0.032

The proportions of heroin opioid overdose related hospitalizations by year were obtained using cross tabulation and the Pearson Chi-Square (χ^2^) test. Significant *p* values ≤ 0.05 at a 95% Confidence Interval. NA: Native American.
